# Pattern of family dynamics and treatment-seeking behaviour of caregivers of under-five children with uncomplicated malaria in a tertiary hospital in Ilesa, southwestern Nigeria

**DOI:** 10.1371/journal.pone.0354019

**Published:** 2026-07-24

**Authors:** Abdulwasiu Akanfe Adegboyega, Abdulakeem Ayanleye Ahmed, Ibrahim Sebutu Bello, Olanrewaju Oloyede Oyegbade, Samuel Ademola Adegoke, Olufemi M. Olubiyi, Temitope Oluwafemi Olajubu, Kolawole Muideen Adewumi, Fatima Adenike Adegoke, Abraham Busola Oluwajuyitan

**Affiliations:** 1 Department of Family Medicine, Obafemi Awolowo University Teaching Hospitals Complex, Ile-Ife, Osun, Nigeria; 2 Department of Family Medicine, Federal University of Health Sciences, Ila-Orangun, Osun, Nigeria; 3 Department of Paediatrics and Child Health, Obafemi Awolowo University, Ile-Ife, Nigeria; 4 Department of Family Medicine, BAFROW Medical Centre, Serrekunda, The Gambia; George Washington University School of Medicine and Health Sciences, UNITED STATES OF AMERICA

## Abstract

**Background:**

The morbidity and possible consequent mortality resulting from malaria in children are either due to delayed treatment-seeking or preceded by an inappropriate treatment-seeking process. This study aimed to assess the relationship between the pattern of family dynamics and treatment-seeking behaviour of caregivers of under-five children with uncomplicated malaria.

**Methods:**

This was a hospital-based cross-sectional study that recruited 350 child-caregiver pairs. An interviewer-administered questionnaire was used for data collection, and it was analysed using the Statistical Package for the Social Sciences (SPSS) software version 25. A p-value of < 0.05 was taken as statistically significant.

**Results:**

The family dynamics of the caregivers revealed that 82.3% of them had strong family support, four-fifths (80.3%) had functional families, and a little over half of them (58.3%) had monthly family incomes that fell below the defined household poverty line. Only 41.7% of the caregivers had appropriate treatment-seeking behaviour. Caregivers from lower-income families had 2.5 times higher odds of exhibiting inappropriate treatment-seeking behaviour compared to those from higher-income families (OR, 2.494; 95% CI, 1.486–4.184; p = 0.001). Caregivers with dysfunctional families were about four times more likely to have inappropriate treatment-seeking behaviour compared to their counterparts with functional families (OR, 3.766; 95% CI, 1.445–9.813, p = 0.007).

**Conclusion:**

Significant inappropriate treatment-seeking behaviour existed among caregivers of under-five children with uncomplicated malaria. Family factors related to inappropriate treatment-seeking behaviour were family income and family functioning. Strengthening the family and counselling on appropriate treatment-seeking is crucial to reducing malaria morbidity and mortality among under-five children.

## Introduction

Malaria is one of the most prevalent tropical diseases in the world [1] and it is caused by *Plasmodium spp* [2]. Most malaria infection in sub-Saharan Africa is caused by *P. falciparum,* which has a high tendency to cause severe malaria and consequent mortality [3]. Under-five children and pregnant women bear the most burden of the disease, including its severe life-threatening forms, compared to adults, owing to their lower immunity [4]. The WHO, in its 2023 World Malaria Report, ranked Nigeria first in the global malaria burden, contributing 26% of the global malaria cases and being responsible for 31% of the disease’s fatalities [5]. Malaria is the most common reason for childhood admission to Nigerian health facilities [6,7]. Iyanam *et al.* in Akwa Ibom State, southern Nigeria, observed that 49.8% of under-five presentations in hospitals were due to malaria and were responsible for most hospital admissions when severe [6].

Despite this burden, delayed and inappropriate treatment-seeking behaviours are still a major public health concern among caregivers of children under five with uncomplicated malaria. In a Zambian study, just 42% of caregivers of children under five sought appropriate medical attention within 24 hours of a fever episode [8]. Nigeria has one of the lowest rates of care-seeking for suspected malaria infections in the world, with less than 20% of all feverish children under the age of five being admitted to healthcare facilities for medical evaluation and parasitological testing [9]. According to Sampson and colleagues, in Imo State, South-Eastern Nigeria, only 18.6% of caregivers of children under five who had a fever engaged in appropriate health-seeking behaviour [10] even though it is recommended that appropriate malaria treatment be sought within 24 hours of the appearance of the first symptom [2,11]. However, most caregivers usually fail to meet this timeline.

Delayed health-seeking for uncomplicated malaria is a risk factor for severe malaria [12]. The persistence of these inappropriate treatment-seeking behaviours jeopardises local and international efforts to lower malaria-related childhood mortality and hinders the achievement of more general public health objectives [13]. The longer the length of treatment delay, the higher the cost of care [14,15]. An average of US$31.49 (₦157) was spent on each case of uncomplicated malaria in research by Ezenduka *et al.* that assessed the treatment expenses for the disease at a public healthcare facility in Nigeria [16]. Not only can this be more with an initial inappropriate effort, but it also strains family finances.

Several studies in different parts of Africa have assessed factors related to the treatment-seeking behaviour of caregivers of under-five children with fever [8,10,17,18]. However, all these studies focused only on sociodemographic, socioeconomic and clinical determinants, overlooking the contribution of family dynamics. There is a scarcity of studies evaluating the influence of family dynamics on treatment-seeking behaviour for febrile under-five children with malaria. Family and its dynamics can also influence the treatment-seeking behaviour of caregivers of an ill child [17]. The family serves as the source of emotional and financial support, and even as a source of making decisions for seeking treatment. Studying the treatment-seeking behaviour of febrile under-five children with malaria with respect to their family dynamics can help reduce treatment delay and improve the prevalence of appropriate treatment-seeking behaviour.

One strategy to address the high incidence and mortality rate of malaria is to investigate the family dynamics and treatment-seeking behaviour of the caregivers of the affected children from the time the fever starts until the proper treatment is started. Undoubtedly, the family has a crucial role in reducing malaria morbidity and mortality in this age range. Its dynamics can alter the amount of time that treatment is delayed and the course of the disease.

The study by Lovelyn *et al.* assessed the family and social determinants of health-seeking behaviour among the caregivers of children with fever [17]. This study was non-specific to malaria and only assessed the influence of family characteristics and family functioning, overlooking the role that family support, which is an essential factor in family dynamics, could have on the treatment-seeking behaviour of under-five children. According to Dagnew *et al.*, the age of the mother and the size of the family all predicted the mothers’ health-seeking behaviour in Ethiopia [19].

The treatment of any child with malaria, like any other disease, is mostly dependent on the family, and the effectiveness of any disease control program is significantly influenced by the family and its dynamics [20] which is the pattern of interaction among members of a family concerning their individual goals and preferences is called family dynamics [21]. Since the children are incapable of expressing the severity of their illness and navigating the healthcare system on their own, parents choose the kind and timing of healthcare services their children receive [22]. This makes seeking medical attention for them a special one. Family support has a big impact on the outcome of any illness [23]. The family, most especially the mother, is the first to notice fever, reduced activity, and other symptoms of malaria in a child. The family give instrumental and financial support that facilitates prompt and appropriate health-seeking attempts [24].

Considering the large burden of malaria among under-five children in Nigeria and the paucity of studies on the influence of family dynamics on the health-seeking behaviour of their caregivers, it is important to study the treatment-seeking behaviour for under-five children with malaria and how it is influenced by family characteristics, family support and family functioning, which are critical factors in the dynamics of families. Such insights could help to reduce delays in diagnosis, initiation of treatment, and supply suggestions for the elimination of malaria. The study attempts to bridge a wide and longstanding gap in knowledge on interactions between family dynamics and the health-seeking behaviour of caregivers of under-five children in Nigeria, the African region, and globally.

This study aimed to assess the relationship between the pattern of family dynamics and treatment-seeking behaviour of caregivers of under-five children with uncomplicated malaria in order to enhance early appropriate treatment-seeking for malaria and reduce malaria-related mortality. The specific objectives were to assess the pattern of family dynamics of caregivers of under-five children with uncomplicated malaria, to describe the pattern of their treatment-seeking behaviour, and to determine the relationship between the pattern of family dynamics and caregivers’ treatment-seeking behaviour for under-five children with uncomplicated malaria.

We hypothesised that there would be a negative association between family dynamics and health-seeking behaviour, whereby caregivers from dysfunctional and non-supportive families would have higher-level risks of inappropriate health-seeking behaviour. This study is based on the health belief model as the primary theoretical framework [25], which links health-seeking behaviour to social constructs such as social support and family dynamics factors. Family dynamics factors such as family functionality, support, and income are related to the health-seeking behaviour of caregivers [26,27].

## Materials and methods

### Study design, study site, and study population

It was a hospital-based cross-sectional analytical study conducted at the Children’s Welfare Clinic of the Wesley Guild Hospital (WGH), Ilesa, Osun State, southwestern Nigeria. WGH is a public tertiary healthcare facility owned by the Obafemi Awolowo University Teaching Hospitals Complex and provides primary, secondary, and tertiary healthcare to the people of Ilesa and the surrounding Ekiti, Edo, Ondo, and Oyo States. Ilesa is an urban municipality located in a region with a high rate of stable malaria transmission, where malaria remains one of the leading causes of outpatient visits, emergency unit admissions, and childhood morbidity.

The hospital provides paediatric healthcare services through the Children’s Welfare Clinic, Children’s Emergency Ward, and various specialist clinics. Children with uncomplicated malaria are managed primarily at the Children’s Welfare Clinic, while those presenting with features of severe malaria are referred promptly to the Children’s Emergency Ward for admission and specialist care. The Children Welfare Clinic, which is staffed by Consultant Paediatricians and resident doctors, handles over 150 under-five children every week from 8:00 am to 4:00 pm, Monday through Friday. According to national recommendations, the diagnosis of malaria is based on malaria rapid diagnostic test or malaria microscopy. Although some patients are covered by the National Health Insurance Authority or other health insurance programs, healthcare services are rendered predominantly on a fee-for-service basis. During the study period, caregivers paid for consultation according to the hospital’s approved rate before receiving treatment, but malaria microscopy was done at no cost.

All children aged 6–59 months with fever diagnosed with uncomplicated malaria using microscopy and their caregivers aged ≥ 18 years who gave written informed consent were eligible to participate in the study. Children having any features of severe malaria like convulsion, altered sensorium, prostration, etc., those whose clinical features necessitate hospital admission, and those whose caregivers are not members of their family were excluded from study participation.

### Sample size and sampling technique

The minimum sample size was determined using the formula [28]:


$$\begin{array}{c} n=\frac{z^{\text{2}}pq}{d^{\text{2}}} \end{array}$$

Where z = standard deviation of 1.96, which corresponds to a 95% confidence interval.

P = 0.29 (published local prevalence of appropriate or good treatment-seeking behaviour for under-five children with fever) [29]


$$\begin{array}{c} \text{q}=\text{1}-\text{p}=\text{0}.\text{71} \end{array}$$

d = Desired level of precision (maximum error of estimate) =0.05%

n = the minimum sample size.

Z = 1.96 at a 95% confidence interval and d = 0.05 at a precision level of 5%.

Using the above formula;


$$\begin{array}{c} \text{n}=\frac{{\text{1}.\text{9}\text{6}}^{\text{2}\;}\times \text{0}.\text{2}\text{9}\times \text{0}.\text{7}\text{1}\;}{{\text{0}.\text{0}\text{5}}^{\text{2}}}\approx \text{317} \end{array}$$

The calculated sample size was adjusted upward to 350 to improve the statistical power and reliability of the study findings. Using a systematic sampling technique, 350 child-caregiver pairs with malaria attending the Children’s Welfare Clinic who met the inclusion criteria were recruited. Hospital records indicated that an average of 60 under-five children with malaria were seen weekly in 2023, giving an estimated sampling frame of 780 patients over the 13-week study period. A sampling interval of two was therefore calculated (780/350 ≈ 2). The first participant was selected by simple random balloting from the first two eligible caregivers, after which every second eligible caregiver was recruited. Where a caregiver declined participation, the next eligible caregiver was selected, and subsequent recruitment continued using the same interval. Approximately 27 participants were enrolled weekly, with five to six recruited daily from Monday to Friday, until the required sample size was attained.

### Data collection

A pretested, structured, interviewer-administered questionnaire (S1 File) was used to obtain data on sociodemographic characteristics of the children and caregivers, caregivers’ treatment-seeking behaviour, and pattern of family dynamics. The study was carried out between March and May 2024. It was administered by trained field workers who were themselves resident doctors with experience in managing malaria in children. They were trained on the study protocol, the use of the questionnaire and blood sample selection. The study instrument was available in English, but it was translated into the Yoruba language for non-English speaking respondents (S2 File). The questionnaire was translated from English to Yoruba by a linguistics expert and back-translated from the Yoruba language to the English language by another independent translator for comparison. The researchers and their trained assistants were present at the clinic every Monday-Friday to screen the children aged 6–59 months for eligibility. History taking and physical examination were conducted by the author. The caregivers of the children were informed of the study, and written informed consent was obtained. Data collected for each caregiver included age, sex, marital status, relationship with the child, occupation, and level of education.

#### Caregivers’ treatment-seeking behaviour.

The caregivers’ treatment-seeking behaviour was assessed using an adapted WHO-validated questionnaire on treatment-seeking behaviour and treatment delay for tuberculosis [30] and it is in line with those used in other similar studies on caregivers’ treatment-seeking behaviour for under-five children with fever in Nigeria. [10,17] It was further validated (face and content) among 35 caregivers of under-five children with fever in another tertiary healthcare facility in Ile-Ife. Treatment-seeking behaviour was dichotomised into either appropriate or inappropriate. Caregivers who sought treatment from a formal healthcare provider within 24 hours of the onset of the child’s illness [10] or had his/her child treated at home by a trained community healthcare worker using pre-packaged antimalarial within 24 hours of the onset of the first malaria symptom, were considered to have appropriate treatment-seeking behaviour [31]. Caregivers who sought treatment from an informal healthcare provider within 24 hours of the child’s illness or from a formal healthcare provider after 24 hours of the child’s illness were considered to have inappropriate treatment-seeking behaviour [10].

#### Pattern of family dynamics.

The caregivers’ family dynamics was assessed through the family characteristics (including the type of family, family income, and family size), perceived family support using the Perceived Social Support Family Scale (PSS-Fa Scale) and perceived family functioning using the Family APGAR score. The caregivers’ monthly family income was dichotomised using the Household Poverty Line as a cut-off into <₦275,000 and ≥ ₦275,000 [32]. The Household Poverty Line was $229, which was equivalent to ₦275,000 at the rate of ₦1200 per dollar between March and May 2024 when the data was collected.

The PSS-Fa, which consists of 20 questions, has a Cronbach’s Alpha coefficient ranging from 0.88 to 0.91, indicating strong validity and reliability [33]. Each “yes” response was scored 1, and “no” or “don’t know” was scored zero. Items iii, iv, xvi, xix and xx were reverse scored: a “no” is scored as 1. Of the total score of 20, a score of ≥ 11 denoted strong family support, scores 7–10 denoted weak family support, while scores ≤ 6 denoted no family support [33]. The PSS-Fa summated scores were used to categorise each respondent’s familial support. Strong family support was indicated by scores of 11 or higher, weak family support by scores of 7–10, and no family support by scores of 6 or lower [34].

The caregivers’ perception of their family functioning was assessed using the Family APGAR, a five-item validated instrument [35]. According to a recent validity analysis of the Family APGAR questionnaire, its Cronbach’s alpha coefficient was 0.819, which denoted good validity and reliability [36]. Each question was scored on a scale of two (almost always), one (some of the time), or zero (hardly ever), and the points were added up, making the lowest score of ‘0’ and the highest score of ‘10’. A score of 0–3 denoted a severely dysfunctional family, 4–6 a moderately dysfunctional family, and 7–10 a highly functional family [37]. The Family APGAR questionnaire and the Perceived Social Support from Family Scale (PSS-Fa) used in this study are provided in Section D of the study questionnaire (S1 and S2 Files).

#### Child characteristics and malaria parasite density.

Data on the child’s age in months, sex, position in the family and malaria parasite density were collected. After briefly playing with the child to allay his/her fear and anxiety, the ball of the thumb that had been cleaned with an alcohol swab was gently pricked with a new, sterile, disposable lancet. While applying gentle pressure, the first drop of blood was expressed and wiped away with a ball of dry cotton wool while ensuring that no cotton strands remained. While holding a labelled microscope glass slide only by the labelled edge and simultaneously applying gentle pressure to the finger, one drop of capillary blood was collected in the centre of the slide, and another drop was collected close to the unlabelled edge of the glass slide. A ball of dry cotton wool was used to wipe off the remaining blood from the finger. Thick film was made with the blood drop located in the centre of the glass slide, and thin film for malaria parasites was made with the other drop. Fixing and staining of the films were done following the World Health Organisation protocol [38] by the study malaria microscopists from the Department of Microbiology and Parasitology of the hospital.

A light microscope was used to examine the blood films under oil immersion at a magnification of ×1000. Estimating asexual parasite density in the thick films was done by first counting the number of asexual parasites with respect to 200 white blood cells (WBC) or counting 500 asexual parasites, whichever occurred first. The malaria parasite density (per μl of blood) was determined using the formula [39]:

$\text{Parasite density }(/\mu \text{l})\;=\frac{\text{ Number of parasites counted}}{\text{Number of leukocytes counted}}\times \text{ 800}$

Two malaria microscopists independently examined each slide, and the average of their readings was taken as the parasite density. A malaria parasitologist reexamined the slide if there was a discrepancy of more than 25% in the parasite density. The final parasite density was determined by averaging the two most consistent counts.

To make sure that no child was recruited more than once, the case note of each recruited child was labelled with the words “MALARIA FAMILY DYNAMICS STUDY.” After the study, these were erased from the case notes.

### Operational definition of terms

For the purpose of this study, families with Family APGAR scores of 7–10 were classified as functional, scores of 4–6 as moderately dysfunctional, and scores of 0–3 as severely dysfunctional. Strong family support was indicated by scores of 11 or higher, weak family support by scores of 7–10, and no family support by scores of 6 or lower. Appropriate treatment-seeking behaviour was defined as seeking care from a recognised health facility within 24 hours of symptom onset, while all other care-seeking patterns were classified as inappropriate.

### Data analysis

Version 25 of the statistical package for the social sciences (SPSS) software (SPSS, Chicago, Il, USA) was used to code, enter and analyse the data. All variables were subjected to descriptive statistics. Descriptive statistics were used to analyse the characteristics of the respondents, including their gender, age group, occupation, ethnicity, education level, and distance to the nearest healthcare facility. While means and standard deviations were used to express continuous data, proportions and percentages were used to express categorical variables.

A cross-tabulation of treatment-seeking behaviours was conducted using the Family APGAR, PSS-Fa, and Family Characteristics, which evaluated family dynamics. A bivariate analysis was conducted using the Chi-square test to evaluate the factors associated with treatment-seeking behaviours. This analysis looked at the relationship between the dependent variables (treatment-seeking behaviour pattern) and the independent variables (caregiver’s family characteristics, PSS-Fa score, family APGAR score, and other family characteristics). Multivariate logistic regression was done to determine the family factors that were independent predictors of health-seeking behaviour. Statistical significance was defined as p-values of less than 0.05 for all statistical analyses. A pie chart and tables were used to display the data.

### Ethical approval

Ethical approval was obtained from the OAUTHC, Ile-Ife ethical review committee, with protocol number ERC/2022/11/18. Following their eligibility screening and prior to the start of data collection, informed consent was obtained from the caregivers of eligible children before they were recruited. Confidentiality of all information was ensured by maintaining anonymity. The questionnaires and the microscopy slide for malaria were identified by assigned study identification numbers. Only the researcher had access to the respondent’s information by keeping all electronic documents password-protected and the hard copies under lock. Each participant retained the right to withdraw from the study at any point in time without any prejudice or penalty.

## Results

**Fig 1:**
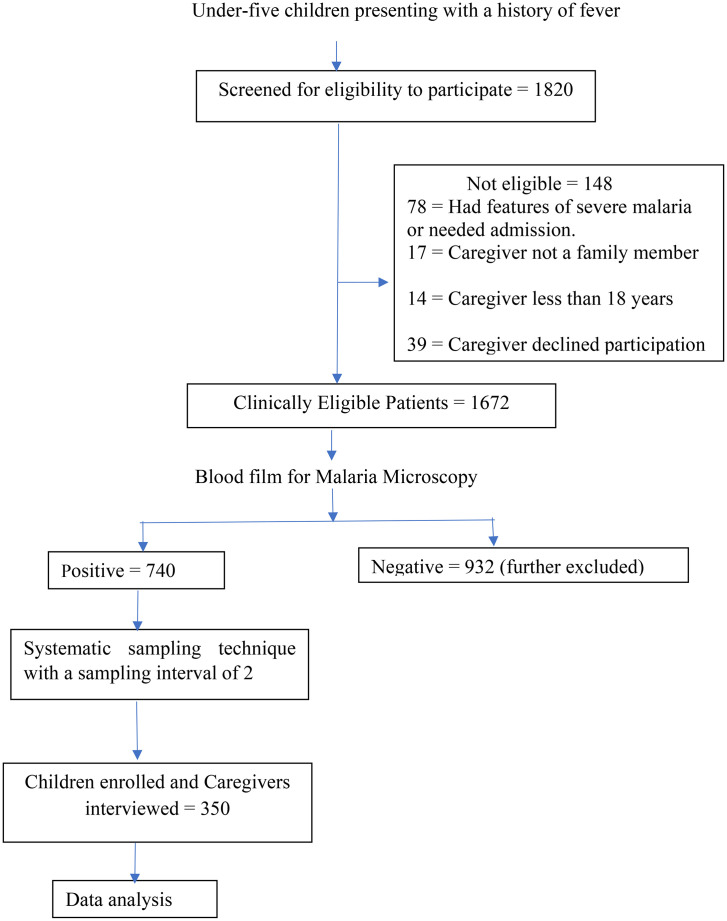
A flow chart showing the study protocol.

A total of 1820 under-five child-caregiver pairs were assessed for eligibility throughout the study period (Fig 1). Of these, 1672 met the inclusion criteria following clinical evaluation, while 148 were excluded because of the presence of exclusion criteria or caregiver refusal of consent. An additional 932 child-caregiver pairs were excluded because they were malaria parasite-negative on microscopy. Only 350 child-caregiver pairs were enrolled following the application of a systematic random sampling technique on the eligible 740 child-caregiver pairs throughout the study duration.

### Baseline characteristics of the study participants

From Table 1 below, it was deduced that the mean age of under-five children with malaria was 30.86 ± 19.00 months, with the modal age group being 6–23 months. The male-to-female ratio among the study participants was 1:1. A larger number of the children were either first-born (36.6%) or second-born (28.9%). Their malaria parasite density ranged from 520 to 17,450 parasite/µL, with a mean parasite density of 8,542.40 ± 7,856.91 parasite/µL.

**Table 1:** Sociodemographic characteristics of under-five children with uncomplicated malaria.

Characteristics	Frequency (N = 350)	Percentage (%)
Age (months)		
6–23	139	39.7
24–41	87	24.9
42–59	124	35.4
Mean ± SD (months)	30.86 **±** 19.00	
Sex		
Male	176	50.3
Female	174	49.7
Child’s Position in the Family		
1^st^	128	36.6
2nd	101	28.9
3^rd^	66	18.9
≥ 4^th^	55	15.7

Table 2 below illustrates the sociodemographic variables of the participants’ caregivers. It was observed that their mean age was 34.55 ± 7.45 years, with 53.4% of them being in the age bracket 25–34 years. In almost all cases, the caregivers were the mothers of the children (91.1%), while fathers accounted for 5.1%, and other caregivers made up 3.8%. In terms of occupation, trading was the most prevalent (47.4%), followed by civil service (22.3%) and artisan work (24.0%). Housewives accounted for a smaller percentage (6.3%). In terms of education, the majority of carers (60.3%) had tertiary education, 35.4% had secondary education, and only 2.3% and 2.0%, respectively, had only a primary education or no formal education. While a small percentage of carers were either never married (1.1%) or had previously been married (2.6%), the vast majority (96.3%) were married. Lastly, 42.6% of the carers lived within 6 km of a health facility, whereas 57.4% of them lived more than 6 km from the closest one.

**Table 2:** Sociodemographic characteristics of caregivers of under-five children.

Characteristics	Frequency (N = 350)	Percentage (%)
Age of caregiver in years		
< 24	9	2.6
25-34	187	53.4
35- 44	128	36.6
≥ 45	26	7.4
Mean ± SD (years)	34.55 ± 7.45	
Marital status		
Never Married	4	1.1
Married	337	96.3
Previously Married*	9	2.6
Relationship with the child		
Mother	319	91.1
Father	18	5.1
Others^#^	13	3.8
Occupation		
Civil Servant	78	22.3
Artisan	84	24.0
Trading	166	47.4
Housewife	22	6.3
Level of Education		
No formal education	7	2.0
Primary	8	2.3
Secondary	124	35.4
Tertiary	211	60.3

* Previously married included divorced and widowed, ^#^ Others included grandmothers and aunts.

### Pattern of family dynamics of the caregivers of under-five children with uncomplicated malaria

Table 3 presents the family dynamics of caregivers of the under-five children with malaria.

**Table 3:** Family dynamics of the children’s caregivers.

Family dynamics variables	Frequency (N = 350)	Percentage (%)
Type of marriage (n = 337)		
Monogamy	331	98.2
Polygamy	6	1.8
*Monthly family income (Naira)		
<₦275,000 ($229)	204	58.3
≥ ₦275,000 ($229)	146	41.7
Family size		
2-6	311	88.9
>6	39	11.1
Perceived Family Support (PSS-Fa)		
Strong family support	288	82.3
Weak family support	45	12.9
No family support	17	4.9
Family functioning (family APGAR)		
Severely dysfunctional family (0–3)	18	5.1
Moderately dysfunctional family (4–6)	51	14.6
Highly functional family (7–10)	281	80.3

*US$1 = ₦1,200 as at the time of conducting the study.

The vast majority (98.2%) of the 337 married caregivers were in monogamous marriages. Just 1.8% of people were in polygamous marriages. A little more than half of the families (58.3%), according to the income distribution, earn less than ₦275,000 ($229) per month. The remaining 41.7% earned at least ₦275,000 ($229) a month.

In terms of family size, 88.9% of the caregivers’ families had two to six members, making them comparatively small. Just 11.1% of the families had more than six people. Strong family support was reported by a sizable percentage of caregivers (82.3%), according to the Perceived Family Support (PSS-Fa) score. Just 4.9% of respondents said they had no family support at all, while a smaller group (12.9%) said they had weak family support. In terms of family functioning, 80.3% of the carers were from very functional families. Just 14.6% of people were moderately dysfunctional, and only 5.1% of people were severely dysfunctional.

### Treatment-seeking behaviour of the caregivers of under-five children with uncomplicated malaria

Table 4 presents an analysis of the treatment-seeking behaviour of the caregivers, focusing on the duration of complaints, the initial source of treatment, the time taken to seek the first source of treatment, the time taken to consult a formal healthcare provider, and the type of formal healthcare facility first utilised. The majority of the children had experienced symptoms for at least 24 hours before their caregivers took action. Specifically, 41.1% of them sought treatment between 24 and 48 hours, and 34.0% sought treatment between 8 and less than 24 hours, while a smaller proportion (18.0%) delayed seeking treatment for three days or more.

**Table 4:** Pattern of treatment-seeking behaviour of the caregivers.

Variables	Frequency (N = 350)	Percentage (%)
Duration of Complaints		
Immediately < 8hours	24	6.9
8 – < 24 hours	119	34.0
2 days (24–48 hours)	144	41.1
≥ 3 days (> 48hours)	63	18.0
The first source of seeking treatment		
At home using self-medication	93	26.6
At a patent medicine vendor	124	35.4
At a traditional healer’s home	2	0.6
At church, mosque/faith homes	2	0.6
At home with the help of a formal healthcare provider using pre-packaged antimalarial	4	1.1
At a community pharmacy store	13	3.7
At the Health facility	112	32.0
Length of time to the first source of seeking treatment		
< 8hours	146	41.7
8 – < 24 hours	129	36.9
24 −48 hours	46	13.1
> 48hours	29	8.3
Length of time to a formal Healthcare provider		
< 8hours	22	6.3
8 – < 24 hours	124	35.4
24 - 48 hours	84	24.0
48 – < 72 hours	80	22.9
72 hours and above	40	11.4
Formal Healthcare facility from which treatment was first sought		
Primary Healthcare Centre	14	4.0
Community pharmacy	9	2.6
Private Hospital	8	2.3
Public Hospital	319	91.1

The caregivers predominantly sought initial treatment from informal sources. Patent medicine vendors were the most common first point of contact, utilised by 35.4% of caregivers. Self-medication at home was also a significant first step for 26.6% of caregivers. Only 29.1% of caregivers went directly to a formal healthcare facility as their first source of treatment. Concerning the length of time to the first source of seeking treatment, a notable portion of caregivers (41.7%) sought their first source of treatment within 8 hours of the complaint. Another 36.9% sought treatment between 8 and less than 24 hours. However, 13.1% waited 24–48 hours, and 8.3% waited over 48 hours.

Despite the quick action to the first source, there was a delay in reaching a formal healthcare provider. The largest group, 35.4%, accessed a formal healthcare provider between 8 and less than 24 hours. This was followed by 24.0% who waited 24–48 hours, and 22.9% who waited 48 to less than 72 hours. A smaller percentage (11.4%) waited 72 hours or more, and only 6.3% reached a formal healthcare provider within 8 hours. With respect to the formal healthcare facility from which treatment was first sought, the vast majority (91.1%) of the caregivers went to a Public Hospital. Other formal healthcare facilities were far less frequently utilized as the first point of contact: Primary Healthcare Centres (4.0%), community pharmacies (2.6%), and private hospitals (2.3%).

**Fig 2:**
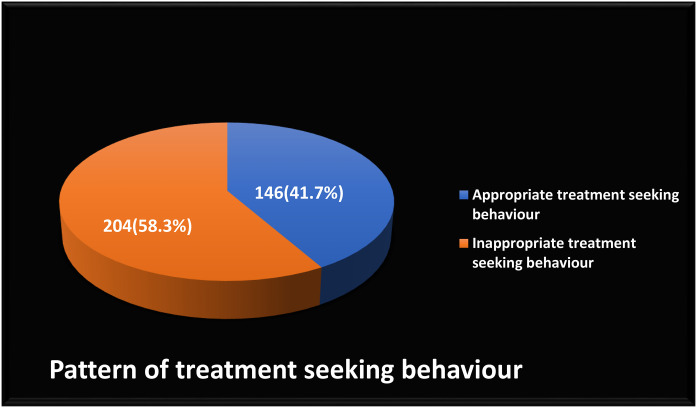
Pattern of treatment-seeking behaviour.

A little over two-fifths (41.7%) of the caregivers exhibited appropriate behaviour when seeking treatment, while a significant majority, nearly three-fifths (58.3%) of the caregivers, displayed inappropriate behaviour in their treatment-seeking actions (Fig 2).

### Relationship between the pattern of family dynamics and caregivers’ treatment-seeking behaviour for under-five children with uncomplicated malaria

Table 5 showed the relationship between the pattern of family dynamics and caregivers’ treatment-seeking behaviour for under-five children with uncomplicated malaria. Being from a polygamous family was significantly associated with a higher tendency to experience inappropriate health-seeking behaviour compared to those who are from monogamous families (n = 13, 81.2%) versus (n = 178, 55.5%) with a p-value of 0.018. More caregivers with a family size greater than six (n = 34, 87.2%) experienced inappropriate health-seeking behaviour compared to those with smaller family sizes (n = 170, 54.7%), with a p-value of 0.001. Inappropriate treatment-seeking behaviour was significantly more prevalent among caregivers earning less than ₦275,000 (n = 144, 70.6%) versus (n = 60, 44.1%) with a p-value of 0.001.

**Table 5:** Relationship between the pattern of family dynamics and caregivers’ treatment-seeking behaviour for under-five children with uncomplicated malaria.

Treatment-Seeking Behaviour					
Variables	Appropriate n(%)	Inappropriate n(%)	Total (N = 350)	χ^2^	P-value
Family Type					
Monogamous	143(44.5)	178(55.5)	321(100)	5.554^+^	0.018*
Polygamous	3(18.8)	13(81.2)	16(100)		
Family size					
2-6	141(45.3)	170(54.7)	311(100)	15.071	0.001*
> 6	5(12.8)	34(87.2)	37(100)		
Monthly family income					
^#^ < ₦275,000	60(29.4)	144(70.6)	204(100)	30.443	0.001*
≥ ₦275,000	86(58.9)	60(41.1)	146(100)		
Family Support					
Strong family support	135(46.9)	153(53.1)	288(100)		
Weak family support	6(13.3)	39(86.7)	45(100)	19.121	0.001*
No family support	5(29.4)	12(70.6)	17(100)		
Family functioning					
Severely dysfunctional	2(11.1)	16(88.9)	18(100)		
Moderately dysfunctional	8(15.7)	43(84.3)	51(100)	26.308^+^	0.001*
Highly functional	136(48.4)	145(51.6)	281(100)		

* Significant at 95% CI, + - Fisher’s exact value

# US$1 = ₦1,200 as at the time of conducting the study.

Inappropriate treatment-seeking behaviour was significantly more prevalent among the caregivers with no family support (n = 12, 70.6%) and those with weak family support (n = 39, 86.7%) compared with those with strong family support (n = 153, 53.1%) (p = 0.001). Inappropriate treatment-seeking behaviour was significantly more prevalent among the caregivers with dysfunctional families, and its prevalence increased with increasing family dysfunctionality as it was (n = 43, 84.3%) and (n = 16, 88.9%) among those with moderate and severe dysfunctional families, respectively (p = 0.001).

Table 6 presents the multiple logistic regression model, which was constructed using all family dynamics characteristics significantly associated with treatment-seeking behaviour in the bivariate analysis to find independent predictors of inappropriate treatment-seeking behaviour among caregivers of children under five. Monthly family income and family functioning were the factors that kept their determinant as independent predictors.

**Table 6:** Multiple logistic regression of the relationship between family dynamics and inappropriate treatment-seeking behaviour among the respondents.

Family dynamics variables	Treatment-seeking behaviour	p-value
	OR (95% Confidence Interval)	
Family Type		
Monogamous	0.518(0.137–1.961)	0.333
Polygamous	1	
Family size		
2 - 6	0.740(0.474–1.156)	0.186
> 6 (ref)	1	
Monthly family income		
^#^ < ₦275,000	2.494(1.486–4.184)	0.001*
≥₦275,000 (ref)	1	
Perceived Family Support (PSS-Fa rating)		
Strong family support	0.655(0.255–1.687)	0.381
Weak/no family support	1	
Family functioning (family APGAR score)		
Dysfunctional family	3.766(1.445–9.813)	0.007*
Functional family (ref)	1	

* Significant at 95% confidence interval; ref: reference category.

# US$1 = ₦1,200 as at the time of conducting the study.

The caregivers who earn less than ₦275,000 monthly were more than twice as likely to have inappropriate treatment-seeking behaviour compared to their counterparts who earn up to and above that (OR, 2.494; 95% CI, 1.486–4.184, p = 0.001). The respondents with dysfunctional families, as assessed by their family APGAR, were about four times more likely to have inappropriate treatment-seeking behaviour compared to their counterparts with functional families (OR, 3.766; 95% CI, 1.445–9.813, p = 0.007). The respondents with strong family support were about 35% less likely to have inappropriate treatment-seeking behaviour compared to their counterparts with no family support (OR, 0.655; 95% CI, 0.255–1.687, p = 0.381).

## Discussion

This study assessed the relationship between the pattern of family dynamics and treatment-seeking behaviour for under-five children with uncomplicated malaria in Wesley Guild Hospital, Ilesa, Nigeria, in order to enhance early and appropriate treatment-seeking for malaria and reduce malaria-related mortality. The family dynamics of the caregivers showed that about four-fifths of them had strong family support, while about four-fifths of them had functional families. Only 41.7% of the caregivers had appropriate treatment-seeking behaviour. The factors that were independently related to reduced likelihood of having inappropriate treatment-seeking behaviour were low monthly family income and having a dysfunctional family. The respondents with dysfunctional families were about four times more likely to have inappropriate treatment-seeking behaviour compared to their counterparts with functional families.

### The pattern of family dynamics of caregivers of the under-five children

Among married caregivers, a startling 98.2% were monogamous. This was the pattern in another study in Osun State, South-Western Nigeria [40]. According to research, urbanisation and changing social values in southwest Nigeria are favouring modern family norms, such as monogamy [41]. Monogamous households may help with better resource allocation and decision-making, two important factors that influence timely care-seeking when a child is ill.

A larger percentage of the caregivers (88.9%) had a family of fewer than six people. The fact that younger families were linked to smaller family sizes and that the majority of study participants were under 44 years old may explain this. This smaller family size may also be due to the above-mentioned prevalent monogamous marriage type. The prevalence of small family units (88.9%) is consistent with Nigeria’s larger demographic shifts, which include a move towards nuclear family structures in peri-urban areas [42]. Smaller families can respond to a child’s illness more quickly and effectively because they are less burdened with intra-family care-giving and financial strain. This was consistent with the findings from other studies on health-seeking behaviour of caregivers of under-five children by Workineh and colleague in northwest Ethiopia, Mitiku and Assefa in West Ethiopia, and Nwaneri and Sadoh in the South-south Region of Nigeria [14,15,29].

The mean family income was ₦226,415.43 or US$188.68, with a larger percentage (58.3%) of the caregivers earning below the household poverty line of ₦275,000 ($229). This reflects intense economic pressure. As of 2022, the Multidimensional Poverty Index (MPI) survey revealed that 133 million people, or 63% of the population, live in multidimensional poverty in Nigeria [43]. According to the report, half (51%) of all impoverished people are children, and two-thirds (67.5%) of children aged 0–17 are multidimensionally poor based on the National MPI. According to recent data from Nigeria and other sub-Saharan nations, financial difficulties continue to be a major obstacle to receiving healthcare, frequently leading to postponed facility visits or a dependence on informal care providers [44]. In many parts of Nigeria—including Ilesa, Osun State—a high proportion of under-five caregivers earn below the household poverty line, as reflected by this study. A greater proportion (82.3%) of the caregivers had strong family support. Similarly, four-fifths (80.3%) of them had functional families. This high presence of family functionality among the respondents was observed in the study of caregivers of febrile under-five children in southeastern Nigeria [17]. It is possible that the high degree of family support and functioning among the study’s respondents stems from the fact that a greater percentage of them were married and valued having family members nearby to provide them with financial and educational support when their kids were ill with malaria. The high reported levels of family support and functionality probably mitigated the negative effects of poverty, even in the face of financial limitations. The use of healthcare by mothers and children is acknowledged to be significantly facilitated by social and familial networks [45]. Caregivers are better equipped to identify illness, plan care, and mobilise resources—even with limited financial resources—when families are supportive and cohesive.

### The pattern of treatment-seeking behaviour for under-five children with uncomplicated malaria

A little over two-fifths (41.7%) of the caregivers exhibited appropriate behaviour when seeking treatment, while a significant majority, nearly three-fifths (58.3%) of the caregivers, displayed inappropriate behaviour in their treatment-seeking actions. This disparity highlights persistent challenges in ensuring timely and effective access to healthcare for vulnerable populations. The prevalence of inappropriate health-seeking behaviour in this study (58.3%) is a significant concern, suggesting that many children may not be receiving the necessary prompt and appropriate medical attention, potentially contributing to higher morbidity and mortality rates.

This finding aligns with that of a community-based survey in Cameroon 47% of caregivers had inappropriate malaria treatment-seeking behaviour [46]. Similarly, a Nigerian study observed that only 18.6% of caregivers had appropriate health-seeking behaviour [10]. Like in this study, both contexts share poor access to reliable healthcare, predominant out-of-pocket payment for healthcare financing, and disruptions to service delivery, influencing timing, choice of provider, and pre-treatment behaviours. On the contrary, a community-based study in Uganda found the prevalence of appropriate health-seeking behaviour to be 69.8% mong the caregivers [47]. This is notably higher than the 41.7% observed in this study. Although both are in sub-Saharan Africa and reflect moderate access to primary healthcare. The probable reasons for the higher rate of appropriate health-seeking behaviour in Uganda are the fact that Wakiso benefits from integrated community case management, ensuring caregivers understand and access prompt care and a strong network of public and private health facilities, with an average travel distance of not more than 3 km, removing geographical barriers.

Regarding the timeliness of seeking care, although only 41.7% of the caregivers sought care for their children within eight hours of the onset of symptoms, more than a third of the caregivers (35.4%) still sought care for their children between 8 and 24 hours after the onset of symptoms. This was close to the report of 53.2% of caregivers of under-five children seeking care for their children within 24 hours of the onset of symptoms in Imo State, Nigeria [10]. This was, however, in contrast to the report by Mitiku and Mekonen [15] where 61.3% of the under-five children’s caregivers sought treatment after 24 hours, just as Hamooya *et al*. [8] also reported that 58% of the caregivers sought care for the under-five children with malaria after 24 hours. This study’s stronger early-care uptake relative to Mitiku and Mekonen and Hamooya *et al.* can be attributed to better malaria awareness and self-efficacy that facilitate timely treatment-seeking attempts**,** improved healthcare access that reduces delays due to distance in Ilesa, being an urban location compared to the rural setting of the Ethiopian and Zambian studies.

### Relationship between the pattern of family dynamics and caregivers’ treatment-seeking behaviour for under-five children with uncomplicated malaria

More respondents with a family size greater than six (87.2%) significantly experienced inappropriate treatment-seeking behaviour compared to those with smaller family sizes (54.7%). Numerous studies have also confirmed the association between larger family sizes and delayed or inappropriate treatment-seeking behaviour [29,48,49]. In particular, a study conducted in Nigeria found that caregivers who had many family members were four times more likely to exhibit negative health-seeking behaviours [10]. This might be the case because larger families are more financially responsible for their family, which could result in greater financial hardship that makes it more challenging to seek early and appropriate treatment. Contrary to the above, some studies have shown that larger family sizes are linked to timely and appropriate treatment-seeking behaviour for children under five who have malaria [10,15]. This is possible because of more family support that may come with a larger size.

Inappropriate treatment-seeking behaviour was significantly more prevalent among participants earning less than ₦275,000 (70.6%) versus (44.1%). This is likely because having a financial constraint makes it difficult to seek timely treatment in a suitable facility, and lower-income carers are also less likely to have health insurance for financing healthcare. This was in tandem with the report of a Myanmar study that cited high treatment and transportation costs as the reason why the majority of the caregivers of under-five children with low family monthly income delayed bringing their children for care [50]. Similarly, Ojewumi and Asaolu discovered that mothers with lower income levels were much more likely to put off getting their under-five children treated for malaria [51]. The relationship between malaria and poverty, as denoted by monthly income, is a bidirectional one. While low income can cause a delay in seeking appropriate healthcare for a child with malaria, as seen above, malaria infection itself can drain the family income and further impoverish the family. Around 36% of low-income families’ monthly income was spent on malaria treatment, compared to 1.2% for wealthier households, according to a 2022 study conducted in Akwa Ibom State that looked at households experiencing malaria episodes [52]. In line with the results of this index study, this underscores the significant financial burden that malaria places on lower-income households.

Wealth may not significantly affect the incidence of malaria, but treatment-seeking behaviour does. Not only are the impoverished more susceptible to contracting malaria, but they also encounter more barriers to receiving official treatment, such as delays brought on by financial constraints and transportation issues. A 2024 qualitative study carried out in Southwest and Northwest Nigeria found that carers, especially the poorest, often turned to patent medicine vendors or herbal remedies for malaria fever due to a combination of factors such as long travel distances, PHC waiting times, poverty and unaffordable facility costs [53]. The earlier finding in this study, where a majority live below the poverty line, echoes this pattern, indicating that financial limitations likely played a role in delays or inappropriate treatment-seeking.

According to earlier research conducted in Nigeria, poverty and malaria are linked in a vicious cycle whereby low income raises the risk of malaria and hinders access to treatment, which further exacerbates poverty. Poor households spend a disproportionate amount of their income on treatment, which increases their financial vulnerability, according to the Akwa Ibom study [52].

In this study, inappropriate health-seeking behaviour was significantly more prevalent among the caregivers with no strong family support (70.6%) and those with weak family support (86.7%) compared with those with strong family support (53.1%). This is because an abundance of family support is essential to the early detection, paying for medical care and keeping follow-up hospital appointments for children with malaria. To corroborate this, Abiodun and Ilori discovered that delayed presentation was linked to dysfunctional parental marital status, which precluded family support in Benin City, Nigeria [54].

Inappropriate health-seeking behaviour was significantly more prevalent among the respondents with dysfunctional families, and its prevalence increased with increasing family dysfunctionality, as it was (84.3%) and (88.9%) among those with moderate and severe dysfunctional families, respectively. A study by Lovelyn *et al.* also revealed a positive correlation between family functionality and the tendency to seek early appropriate treatment for children [17]. When a family is functioning effectively, it can provide for the needs of its members. The time between the onset of malarial symptoms and the diagnosis of malaria is significantly impacted by the likelihood that a patient from a functional family will be able to use family members or have the time for all the required financial, emotional, and instrumental support to present promptly for malaria diagnosis and treatment. A dysfunctional family will be unable to provide its members with timely, appropriate health care and support.

### Relationship between the pattern of family dynamics and caregivers’ treatment-seeking behaviour for under-five children with uncomplicated malaria

More respondents with a family size greater than six (87.2%) significantly experienced inappropriate treatment-seeking behaviour compared to those with smaller family sizes (54.7%). Numerous studies have also confirmed the association between larger family sizes and delayed or inappropriate treatment-seeking behaviour [29,48,49]. In particular, a study conducted in Nigeria found that caregivers who had many family members were four times more likely to exhibit negative health-seeking behaviours [10]. This might be the case because larger families are more financially responsible for their family, which could result in greater financial hardship that makes it more challenging to seek early and appropriate treatment. Contrary to the above, some studies have shown that larger family sizes are linked to timely and appropriate treatment-seeking behaviour for children under five who have malaria [10,15]. This is possible because of more family support that may come with a larger size.

Inappropriate treatment-seeking behaviour was significantly more prevalent among participants earning less than ₦275,000 (70.6%) versus (44.1%). This is likely because having a financial constraint makes it difficult to seek timely treatment in a suitable facility, and lower-income carers are also less likely to have health insurance for financing healthcare. This was in tandem with the report of a Myanmar study that cited high treatment and transportation costs as the reason why the majority of the caregivers of under-five children with low family monthly income delayed bringing their children for care [50]. Similarly, Ojewumi and Asaolu discovered that mothers with lower income levels were much more likely to put off getting their under-five children treated for malaria [51]. The relationship between malaria and poverty, as denoted by monthly income, is a bidirectional one. While low income can cause a delay in seeking appropriate healthcare for a child with malaria, as seen above, malaria infection itself can drain the family income and further impoverish the family. Around 36% of low-income families’ monthly income was spent on malaria treatment, compared to 1.2% for wealthier households, according to a 2022 study conducted in Akwa Ibom State that looked at households experiencing malaria episodes [52]. In line with the results of this index study, this underscores the significant financial burden that malaria places on lower-income households.

Wealth may not significantly affect the incidence of malaria, but treatment-seeking behaviour does. Not only are the impoverished more susceptible to contracting malaria, but they also encounter more barriers to receiving official treatment, such as delays brought on by financial constraints and transportation issues. A 2024 qualitative study carried out in Southwest and Northwest Nigeria found that carers, especially the poorest, often turned to patent medicine vendors or herbal remedies for malaria fever due to a combination of factors such as long travel distances, PHC waiting times, poverty and unaffordable facility costs [53]. The earlier finding in this study, where a majority live below the poverty line, echoes this pattern, indicating that financial limitations likely played a role in delays or inappropriate treatment-seeking.

According to earlier research conducted in Nigeria, poverty and malaria are linked in a vicious cycle whereby low income raises the risk of malaria and hinders access to treatment, which further exacerbates poverty. Poor households spend a disproportionate amount of their income on treatment, which increases their financial vulnerability, according to the Akwa Ibom study [52].

In this study, inappropriate health-seeking behaviour was significantly more prevalent among the caregivers with no strong family support (70.6%) and those with weak family support (86.7%) compared with those with strong family support (53.1%). This is because an abundance of family support is essential to the early detection, paying for medical care and keeping follow-up hospital appointments for children with malaria. To corroborate this, Abiodun and Ilori discovered that delayed presentation was linked to dysfunctional parental marital status, which precluded family support in Benin City, Nigeria [54].

Inappropriate health-seeking behaviour was significantly more prevalent among the respondents with dysfunctional families, and its prevalence increased with increasing family dysfunctionality, as it was (84.3%) and (88.9%) among those with moderate and severe dysfunctional families, respectively. A study by Lovelyn *et al.* also revealed a positive correlation between family functionality and the tendency to seek early appropriate treatment for children [17]. When a family is functioning effectively, it can provide for the needs of its members. The time between the onset of malarial symptoms and the diagnosis of malaria is significantly impacted by the likelihood that a patient from a functional family will be able to use family members or have the time for all the required financial, emotional, and instrumental support to present promptly for malaria diagnosis and treatment. A dysfunctional family will be unable to provide its members with timely, appropriate health care and support.

## Limitations

The study is, however, limited by its cross-sectional design; thus, it can only suggest an association and not a cause-and-effect relationship between the pattern of family dynamics and treatment-seeking behaviour. Secondly, information obtained through an interviewer-administered questionnaire may result in underreporting of poor treatment-seeking behaviour and family characteristics, and this may affect the outcome. Also, other potential factors, such as health literacy, severity of symptoms at onset, cultural health beliefs, and health system factors, such as distance to the health facility, transportation challenges, or waiting time before receiving care, that could influence treatment-seeking behaviour were not assessed. Moreover, while the quantitative approach used in this study revealed the relationship between caregivers’ patterns of family dynamics and treatment-seeking behaviour for under-five children with uncomplicated malaria, it does not deeply explore motivations or barriers influencing the treatment-seeking behaviour of the caregivers, as a qualitative approach would have done.

Furthermore, this study’s inclusion of solely caregivers of children with laboratory-confirmed uncomplicated malaria is one of its limitations. As a result, the results might not apply to caregivers of children who have febrile illnesses that are not caused by malaria. It would have been possible to evaluate whether family dynamics affect treatment-seeking behaviour regardless of the underlying cause of fever and to compare malaria-positive and malaria-negative febrile illnesses directly if a comparison group of children with negative malaria test results had been included. Future studies using a test-negative or all-cause febrile illness design may provide deeper insights on caregiver treatment-seeking behaviour.

## Conclusion

Although most caregivers reported strong family support and functional family structures, fewer than half demonstrated appropriate treatment-seeking behaviour, indicating that family functionality did not translate into optimal health-seeking practices. Self-medication and patronage of patent medicine vendors were the most common initial responses to childhood illness. Appropriate treatment-seeking was associated with low family income, and dysfunctional family structure was linked to inappropriate health-seeking behaviour.

The findings of this study highlight the importance of family dynamics as a determinant of caregivers’ treatment-seeking behaviour for under-five children with uncomplicated malaria. Family-centred health education and counselling, and routine assessment of family functioning in paediatric and primary healthcare settings may help promote appropriate treatment-seeking behaviour. Integrating these approaches into existing malaria control and child health programmes could contribute to improved health outcomes among under-five children.

## Supporting information

S1 File English version of questionnaire. (DOCX)

S2 File Yoruba version of questionnaire. (XLSX)

S3 File Data set. (XLSX)

S4 File Data dictionary codebook. (DOCX)
