# Supplementary material for: Pattern of family dynamics and treatment-seeking behaviour of caregivers of under-five children with uncomplicated malaria in a tertiary hospital in Ilesa, southwestern Nigeria
Source: PLoS One. 2026 Jul 24;21(7):e0354019. doi: 10.1371/journal.pone.0354019 (PMC13399444; doi:10.1371/journal.pone.0354019)
Supplement: S1 File — (DOCX) [file pone.0354019.s001.docx]

#

**PATTERN OF FAMILY DYNAMICS AND TREATMENT-SEEKING BEHAVIOUR OF CAREGIVERS OF UNDER-FIVE CHILDREN WITH UNCOMPLICATED MALARIA IN A TERTIARY HOSPITAL IN ILESA, SOUTHWESTERN NIGERIA**

**ENGLISH QUESTIONNAIRE**

Good day, Sir/Ma. This research work is aimed at determining the relationship between the pattern of family dynamics and treatment-seeking of caregivers of under-five children with uncomplicated malaria to enhance early appropriate care-seeking and reduce malaria-related mortality. Please answer the following questions truthfully. Your answers will be kept strictly confidential. Thanks for your cooperation.

| Serial No………………………………. |
| --- |

SECTION A – SOCIODEMOGRAPHIC CHARACTERISTICS OF CAREGIVERS AND CHARACTERISTICS OF CHILDREN WITH UNCOMPLICATED MALARIA

1. SOCIODEMOGRAPHIC CHARACTERISTICS OF THE CAREGIVERS

| S/N | Questions and Options (Interviewer should circle the appropriate option. Interviewer should not write in the shaded portion) |  |  |
| --- | --- | --- | --- |
|  |  | Q S/N | Code |
| 1 | What is your age (as of last birthday)? | Q1 |  |
| 2 | What is your relationship with this child?  1=Mother 2=Father 3=Grandmother 4= Elder sibling 5= Aunt 6= Others(Specify…………) | Q2 |  |
| 3 | What is your marital status? 1=Single 2=Married 3=Divorced 4=Widowed 5=Separated | Q3 |  |
| 4 | What is your religion?  1 = Islam 2= Christianity 3= Traditional 4=Others (specify} | Q4 |  |
| 5 | What is your tribe?  1= Yoruba 2 = Hausa 3= Igbo 4=Others (specify) | Q5 |  |
| 6 | What is your occupation? | Q6 |  |
| 7 | How far in Kilometre is your resident from the nearest health facility? | Q7 |  |
| 8 | What is your Highest level of education?  0= None 1= Primary 2= Secondary 3= Tertiary 4= Postgraduate | Q8 |  |

1. ASSESSMENT OF CHARACTERISTICS OF CHILDREN WITH UNCOMPLICATED MALARIA

| S/N | Variables | Q S/N | CODE |
| --- | --- | --- | --- |
| 1 | How old is this ill child in months ?……………… | Q1 |  |
| 2 | What is the gender of this child? ……………………… | Q2 |  |
| 3 | What is the position of this ill child in the family?………... | Q3 |  |
| 4 | How many siblings does the ill child have? ……………….. | Q4 |  |

SECTION B – ASSESSMENT OF CAREGIVER’S KNOWLEDGE OF MALARIA AND MALARIA-RELATED IDEATIONAL FACTORS.

1. ASSESSMENT OF KNOWLEDGE OF MALARIA

| S/N |  | YES | NO | I DON’T KNOW |
| --- | --- | --- | --- | --- |
| 1 | Malaria can be transmitted by mosquito? |  |  |  |
| 2 | Malaria is a disease transmitted by evil spirit? |  |  |  |
| 3 | Malaria is caused by excessive exposure to sunlight? |  |  |  |
| 4 | Can malaria result from physical contact with someone with malaria? |  |  |  |
| 5 | Malaria is more serious in children than adults? |  |  |  |
| 6 | Malaria can be prevented? |  |  |  |
| 7 | Fever is a symptom of malaria? |  |  |  |
| 8 | Vomiting is a symptom of malaria? |  |  |  |
| 9 | Anaemia is a symptom of severe/complicated malaria? |  |  |  |
| 10 | Convulsion is a symptom of severe/complicated malaria? |  |  |  |
| 11 | Malaria needs to be treated immediately? |  |  |  |
| 12 | Traditional herbs/medicine are a good way to treat malaria? |  |  |  |
|  | Total score |  |  |  |

Summary of knowledge of malaria: Good or Poor

1. ASSESSMENT OF PERCEIVED SUSCEPTIBILITY TO MALARIA

| S/N | Variable | Strongly agree | Agree | Disagree | Strongly disagree |
| --- | --- | --- | --- | --- | --- |
| 1 | Malaria is prevalent in your area |  |  |  |  |
| 2 | Fever could be due to malaria |  |  |  |  |
| 3 | Refusal of meal could be due to malaria |  |  |  |  |
| 4 | Vomiting and diarrhoea could be due to malaria |  |  |  |  |
| 5 | Malaria can cause anaemia |  |  |  |  |
| 6 | Children always have a chance to be infected by malaria |  |  |  |  |
|  | Total score |  |  |  |  |

Summary of perceived susceptibility to malaria: high or low

1. ASSESSMENT OF PERCEIVED SEVERITY OF MALARIA

| S/N | Variable | Strongly agree | Agree | Disagree | Strongly disagree |
| --- | --- | --- | --- | --- | --- |
| 1 | Malaria is a serious disease in children |  |  |  |  |
| 2 | Worried that my child is suffering from malaria |  |  |  |  |
| 3 | Complications of malaria are dangerous and can result in death |  |  |  |  |
| 4 | Risk of death is higher in children compared to adults |  |  |  |  |
| 5 | Malaria treatment costs more money if complicated |  |  |  |  |
|  | Total score |  |  |  |  |

Summary of perceived severity of malaria: high or low

1. ASSESSMENT OF PERCEIVED BENEFIT OF MALARIA TREATMENT-SEEKING

| S/N | Variable | Strongly agree | Agree | Disagree | Strongly disagree |
| --- | --- | --- | --- | --- | --- |
| 1 | The child will get well as soon as he/she is brought to the health facility |  |  |  |  |
| 2 | Seeking treatment avoids additional cost to treat complications |  |  |  |  |
| 3 | Seeking treatment reduces the chance of death |  |  |  |  |
| 4 | Value money spent for child treatment-seeking |  |  |  |  |
|  | Total score |  |  |  |  |

Summary of perceived benefit for malaria treatment-seeking: high or low

1. ASSESSMENT OF PERCEIVED BARRIERS TO TREATMENT-SEEKING

| S/N | Variable | Strongly agree | | agree | | Disagree | | Strongly disagree | |  |
| --- | --- | --- | --- | --- | --- | --- | --- | --- | --- | --- |
| 1 | Drugs are not effective to treat malaria | |  | |  | |  | |  | |
| 2 | Malaria subsides by itself without treatment | |  | |  | |  | |  | |
| 3 | Health facility is far from where we live | |  | |  | |  | |  | |
| 4 | Have no money to take child to health facility | |  | |  | |  | |  | |
| 5 | Traditional healers can treat the child with malaria | |  | |  | |  | |  | |
| 6 | The disease is not serious enough | |  | |  | |  | |  | |
| 7 | Home treatment is sufficient | |  | |  | |  | |  | |
| 8 | There will be long waiting time at health at health facility | |  | |  | |  | |  | |
|  | Total score | |  | |  | |  | |  | |

Summary of perceived barrier to treatment: high or low

SECTION C- ASSESSMENT OF PATTERN OF TREATMENT-SEEKING BEHAVIOUR

1. TREATMENT-SEEKING BEHAVIOUR

| S/N | VARIABLES | Q S/N | CODE |
| --- | --- | --- | --- |
| 1 | Child’s presenting symptoms   1. Fever 1=yes 0=no 2. Vomiting 1=yes 0=no 3. Loss of appetite or refusal of meal 1=yes 0=no 4. Headache 1=yes 0=no 5. Joint pain 1=yes 0=no 6. Abdominal pain 1=yes 0=no 7. Weakness or reduced activities 1=yes 0=no 8. Others (specify)…………………………………………. |  |  |
| 2 | Which of the above stated symptoms started first?  ………………………………………………… |  |  |
| 3 | When did the symptom mention in ‘2’ above started?   1. Today < 8hours 2. Today= 8hours – 24 hours 3. Yesterday > 24ours 4. 2 Days ago 5. 3 Days ago 6. 4 Days ago or more |  |  |
| 4 | Where was the first place you sought treatment for your child? : Note- mark only one option here  1= At home using herbs  2= At home using drugs from previous treatment or self-medication  3= At Patent medicine vendor  4= At traditional healer’s home  5= At church, mosque/ faith homes  6= At home with the help of a formal healthcare provider using pre-package antimalarial  7= At a community pharmacy store  8= At health centre  9= At private Hospital  10= At Study centre[Wesley Guild Hospital,(WGH)]  11= At any other government hospital | Q1 |  |
| 5 | How long from the onset of the first symptom mention in ‘2’ above did you take to seek the first treatment marked in ‘4’ above  1= Immediately(less than 8hours)  2=The same day(8hours to 24hours)  3=The second day (24hours to 48hours)  4=The Third day (48hours to 72hours)  5= The fourth day and above (72hours and above) | Q2 |  |
| 6 | When did you seek the care of a formal healthcare provider ( Primary healthcare workers/healthcare provider in a community pharmacy, primary healthcare centre, private hospital, public hospital)?  1= Immediately(less than 8hours)  2=The same day(8hours to 24hours)  3=The second day (24hours to 48hours)  4=The Third day (greater than 48hours to 72hours)  5= The fourth day and above (72hours and above) | Q3 |  |
| 7 | What was the healthcare facility of the formal healthcare provider from whom you first sought care?  1= Primary Healthcare Centre  2= Community Pharmacy  3= Private Hospital  4= Public Hospital |  |  |

SUMMARY OF THE PATTERN OF TREATMENT-SEEKING BEHAVIOUR

| Appropriate treatment-seeking behaviour |  |
| --- | --- |
| Inappropriate treatment seeking behaviour |  |

SECTION D – ASSESSMENT OF PATTERN OF FAMILY DYNAMICS

I. ASSESSMENT OF THE FAMILY CHARACTERISTICS

1. What type is your family? i) Monogamous [ ] ii) Polygamous [ ] iii) Single parent [ ]

2. How many persons are in your family?……………..

3. What is your family income per month (average)? N……………..

II. ASSESSMENT OF PERCEPTION OF FAMILY SUPPORT (PSS-Fa Scale)

Please tick your response to the questions below:

| S/N | Questions | Yes | NO | Don’t know |
| --- | --- | --- | --- | --- |
| 1. | My family gives me the moral support I need. |  |  |  |
| 2. | I get good ideas about how to do things or make things from my family |  |  |  |
| 3. | Most other people are closer to their family than I am |  |  |  |
| 4. | When I confide in the members of my family who are closest to me, I get the idea that it makes them uncomfortable. |  |  |  |
| 5. | My family enjoys hearing about what I think. |  |  |  |
| 6. | Members of my family share many of my interests |  |  |  |
| 7. | Certain members of my family come to me when they have problems or need advice. |  |  |  |
| 8. | I rely on my family for emotional support. |  |  |  |
| 9. | There is a member of my family I could go to if I were just feeling down, without feeling funny about it later. |  |  |  |
| 10 | My family and I are very open about what we think about things |  |  |  |
| 11 | My family is sensitive to my personal needs |  |  |  |
| 12 | Members of my family come to me for emotional support |  |  |  |
| 13 | Members of my family are good at helping me solve problems |  |  |  |
| 14 | I have a deep sharing relationship with a number of members of my family. |  |  |  |
| 15 | Members of my family get good ideas about how to do things or make things from me. |  |  |  |
| 16 | When I confide in members of my family, it makes me uncomfortable |  |  |  |
| 17 | Members of my family seek me out for companionship |  |  |  |
| 18 | I think that my family feels that I'm good at helping them solve problems. |  |  |  |
| 19 | I don't have a relationship with a member of my family that is as close as other people's relationships with family members |  |  |  |
| 20 | I wish my family were much different |  |  |  |

PSS-Fa Score………………

III. ASSESSMENT OF PERCEPTION OF FAMILY FUNCTIONING (Using Family

APGAR scale)

Please tick your response to the questions below:

|  | Family Functioning Questions | Almost always (2) | Some of the time (1) | Hardly ever (0) |
| --- | --- | --- | --- | --- |
| 1. | I am satisfied that I can turn to my family for help when something is troubling me |  |  |  |
| 2. | I am satisfied with the way my family takes things over with me and shares my problems |  |  |  |
| 3. | I am satisfied that my family accepts and supports my wishes to take on new activities |  |  |  |
| 4. | I am satisfied with the way my family expresses affection and responds to my emotions |  |  |  |
| 5. | I am satisfied with the way my family and I share time together |  |  |  |

Family Apgar Score……….
