# Supplementary material for: Pattern of family dynamics and treatment-seeking behaviour of caregivers of under-five children with uncomplicated malaria in a tertiary hospital in Ilesa, southwestern Nigeria
Source: PLoS One. 2026 Jul 24;21(7):e0354019. doi: 10.1371/journal.pone.0354019 (PMC13399444; doi:10.1371/journal.pone.0354019)
Supplement: S4 File — (DOCX) [file pone.0354019.s004.docx]

# S4 File. Data Dictionary (Codebook)

**Variable naming convention:** Variables were named according to the study questionnaire. 'SECTA', 'SECTB', 'SECTC', etc. denote questionnaire Sections A, B, C, etc., while 'Q' denotes the corresponding question number. For example, SECTAQ1 represents Section A, Question 1 of the questionnaire.

## Variable Codebook

| Variable name | Questionnaire location | Variable description | Coding |
| --- | --- | --- | --- |
| SECTSQ1 | SECTION D – ASSESSMENT OF PATTERN OF FAMILY DYNAMICS, Question 1 | 1. What type is your family? i) Monogamous [ ] ii) Polygamous [ ] iii) Single parent [ ] | Refer to questionnaire coding / dataset values |
| SECTSQ2 | SECTION D – ASSESSMENT OF PATTERN OF FAMILY DYNAMICS, Question 2 | 2. How many persons are in your family?…………….. | Refer to questionnaire coding / dataset values |
| SECTSQ3 | SECTION D – ASSESSMENT OF PATTERN OF FAMILY DYNAMICS, Question 3 | 3. What is your family income per month (average)? N…………….. | Refer to questionnaire coding / dataset values |
