# Supplementary material for: Pattern of family dynamics and treatment-seeking behaviour of caregivers of under-five children with uncomplicated malaria in a tertiary hospital in Ilesa, southwestern Nigeria
Source: PLoS One. 2026 Jul 24;21(7):e0354019. doi: 10.1371/journal.pone.0354019 (PMC13399444; doi:10.1371/journal.pone.0354019)
Supplement: Attachment — Submitted filename: Response to Reviewers.docx [file pone.0354019.s006.docx]

**EDITOR**

**Thank you so much for your painstaking effort in reviewing our manuscript. Below are the comments to each of the points raised.**

| **SECTION** | **EDITOR’S COMMENTS** | **AUTHORS’ CORRECTION** |
| --- | --- | --- |
| 1. | Please ensure that your manuscript meets PLOS ONE's style requirements, including those for file naming. The PLOS ONE style templates can be found at [https://journals.plos.org/plosone/s/file?id=wjVg/PLOSOne_formatting_sample_main_body.pdf](https://track.editorialmanager.com/CL0/https:%2F%2Fjournals.plos.org%2Fplosone%2Fs%2Ffile%3Fid=wjVg%2FPLOSOne_formatting_sample_main_body.pdf/1/010f019ea67bdc0f-e78a4a98-7f06-42d3-81d0-922ebf9bc4af-000000/nEajTF6BOwzEvQyMlkd9g58R7UU9U0uW3rwCjcDE5uI=258) and [https://journals.plos.org/plosone/s/file?id=ba62/PLOSOne_formatting_sample_title_authors_affiliations.pdf](https://track.editorialmanager.com/CL0/https:%2F%2Fjournals.plos.org%2Fplosone%2Fs%2Ffile%3Fid=ba62%2FPLOSOne_formatting_sample_title_authors_affiliations.pdf/1/010f019ea67bdc0f-e78a4a98-7f06-42d3-81d0-922ebf9bc4af-000000/hxHh6bLnYfKx_iBOn9HgfEHUjZ6576Lc1xkcLZ2Zcew=258) | The manuscript has been revised to conform to the PLOS ONE formatting and file-naming requirements. All files have been renamed and formatted in accordance with the journal's guidelines. |
| 2. | Your ethics statement should only appear in the Methods section of your manuscript. If your ethics statement is written in any section besides the Methods, please delete it from any other section. | We have reviewed the manuscript and ensured that the ethics statement appears only in the **Methods** section, in accordance with PLOS ONE's requirements. |
| 3. | Please amend either the title on the online submission form (via Edit Submission) or the title in the manuscript so that they are identical. | We have revised the title in the manuscript and the online submission system to ensure that they are identical, in accordance with the journal's requirements. |
| 4. | [Please update your submission to use the PLOS LaTeX template. The template and more information on our requirements for LaTeX submissions can be found at http://journals.plos.org/plosone/s/latex.](http://track.editorialmanager.com/CL0/file:%2F%2F%2FC:%2FUsers%2Fe759388%2FDocuments%2F2026%2FMS%25202026%2FPlease%2520update%2520your%2520submission%2520to%2520use%2520the%2520PLOS%2520LaTeX%2520template.%2520The%2520template%2520and%2520more%2520information%2520on%2520our%2520requirements%2520for%2520LaTeX%2520submissions%2520can%2520be%2520found%2520at%2520http:%2Fjournals.plos.org%2Fplosone%2Fs%2Flatex./1/010f019ea67bdc0f-e78a4a98-7f06-42d3-81d0-922ebf9bc4af-000000/tGinXvVc46ftXJLvpq_OB1zFDfT7dJCSq0PBcBuM38A=258) | Thank you for this information. Our manuscript was prepared and submitted using **Microsoft Word (.docx)**, which is an accepted submission format for PLOS ONE. We have therefore retained the manuscript in Microsoft Word for the revised submission. |
| 5. | We note that Figure S2 in your submission contain copyrighted images. All PLOS content is published under the Creative Commons Attribution License (CC BY 4.0), which means that the manuscript, images, and Supporting Information files will be freely available online, and any third party is permitted to access, download, copy, distribute, and use these materials in any way, even commercially, with proper attribution. For more information, see our copyright guidelines: <http://journals.plos.org/plosone/s/licenses-and-copyright>.  We require you to either (1) present written permission from the copyright holder to publish these figures specifically under the CC BY 4.0 license, or (2) remove the figures from your submission: | We thank the Editor for bringing this to our attention. The file identified contained a copy of the institutional ethics approval certificate issued by our institution. To comply with the journal's copyright requirements under the CC BY 4.0 licence, we have removed this document from the Supporting Information files. The ethics approval is fully described in the Methods section of the manuscript, including the name of the ethics committee and protocol number. |
| 6. | We notice that your supplementary [figures] are included in the manuscript file. Please remove them and upload them with the file type 'Supporting Information'. Please ensure that each Supporting Information file has a legend listed in the manuscript after the references list. | We have removed all supplementary figures from the main manuscript and uploaded them separately as Supporting Information files. In addition, legends for all Supporting Information files have been included at the end of the manuscript after the References, in accordance with the PLOS ONE guidelines. |
| 7. | [Please include captions for your Supporting Information files at the end of your manuscript, and update any in-text citations to match accordingly. Please see our Supporting Information guidelines for more information: http://journals.plos.org/plosone/s/supporting-information.](http://track.editorialmanager.com/CL0/file:%2F%2F%2FC:%2FUsers%2Fe759388%2FDocuments%2F2026%2FMS%25202026%2FPlease%2520include%2520captions%2520for%2520your%2520Supporting%2520Information%2520files%2520at%2520the%2520end%2520of%2520your%2520manuscript,%2520and%2520update%2520any%2520in-text%2520citations%2520to%2520match%2520accordingly.%2520Please%2520see%2520our%2520Supporting%2520Information%2520guidelines%2520for%2520more%2520information:%2520http:%2Fjournals.plos.org%2Fplosone%2Fs%2Fsupporting-information.%2520/1/010f019ea67bdc0f-e78a4a98-7f06-42d3-81d0-922ebf9bc4af-000000/ASK7V7g2SX4t6ZylRHLptDkbg0UUu_R2TrEgq0V_Xv8=258) | We have revised the manuscript accordingly. The participant flowchart and the figure showing the distribution of caregivers according to family type have been incorporated into the main manuscript as **Figure 1** and **Figure 2**, respectively, as they present the study findings. The Supporting Information now comprises only the study questionnaires, uploaded as **S1 File (English version)** and **S2 File (Yoruba version).** Legends for these Supporting Information files have been added at the end of the manuscript after the References, and all in-text citations have been updated to correspond with the revised file numbering, in accordance with the PLOS ONE guidelines |
| 8. | We are unable to open your Supporting Information file [File Name]. Please kindly revise as necessary and re-upload. | Thank you for bringing this to our attention. We have reviewed the Supporting Information files, ensured that they open correctly and are in an appropriate file format, and re-uploaded them. The revised Supporting Information files comprise **S1 File (English version of the study questionnaire) and S2 File (Yoruba version of the study questionnaire)** |
| 9. | Thank you for providing your underlying data as Supporting Information.  We note that the data set contains text or data that is not in English. Please note that PLOS is an English-language publisher, so we require data sets to be provided in English as well. Please upload an English-language version of your data set.  This will also allow us to determine if your data follows PLOS standards per our Data Availability policy here: <https://journals.plos.org/plosone/s/data-availability> | The underlying dataset was originally prepared in SPSS and all variable names and data entries were recorded in English. To facilitate accessibility and comply with the journal's requirements, we have exported the dataset to Microsoft Excel (.xlsx), verified that all variable names, labels, and text entries are in English, and re-uploaded the revised dataset as Supporting Information. |
| 10. | If the reviewer comments include a recommendation to cite specific previously published works, please review and evaluate these publications to determine whether they are relevant and should be cited. There is no requirement to cite these works unless the editor has indicated otherwise. | We carefully reviewed the reviewers' comments and confirmed that no specific publications were recommended for citation. Accordingly, no additional references were added in response to this requirement. |
| 11. | Please review your reference list to ensure that it is complete and correct. If you have cited papers that have been retracted, please include the rationale for doing so in the manuscript text, or remove these references and replace them with relevant current references. Any changes to the reference list should be mentioned in the rebuttal letter that accompanies your revised manuscript. If you need to cite a retracted article, indicate the article’s retracted status in the References list and also include a citation and full reference for the retraction notice. | We have carefully reviewed the reference list to ensure that it is complete, accurate, and consistent with the in-text citations. We also verified that none of the cited articles have been retracted. No References was added during the revision process. |

**REVIEWER 1**

**REVIEWER’S COMMENTS AND AUTHORS’ CORRECTIONS**

**Thank you so much for your painstaking effort in reviewing our manuscript. Below are the comments to each of the points raised.**

| **SECTION** | **REVIEWER’S COMMENTS** | **AUTHORS’ CORRECTION** |
| --- | --- | --- |
| 1. | The study setting is not sufficiently described. It would be helpful to clarify whether the health facility is located in a rural or urban area, is the area high endemic or low, and whether it is publicly or privately owned.  Also how malaria treatment service is provided-either freely accessible or with some consultation fee etc...(also patient trajectory in the event a referal is needed..if such health system info is available). | Thank you for this valuable suggestion. We have expanded the description of the study setting in the Methods section to include the location of the hospital (urban), malaria endemicity of the study area (stable high transmission), ownership of the hospital (public tertiary hospital under Obafemi Awolowo University Teaching Hospitals Complex), the financing of malaria services (fee-for-service with health insurance coverage for some patients), and the referral pathway for children requiring higher levels of care. These additions provide better context for readers regarding the healthcare system within which the study was conducted. |
| 2. | The study population includes only participants with a positive malaria test. The authors may wish to discuss how the findings might differ if individuals with negative test results were also included (e.g., as a test-negative comparison group), particularly in relation to general health-seeking behavior. | The objective of our study was to investigate the relationship between family dynamics and treatment-seeking behaviour among caregivers of under-five children with **laboratory-confirmed uncomplicated malaria**. Consequently, only children with parasitologically confirmed malaria were included to ensure a homogeneous study population and to minimise outcome misclassification. We agree that inclusion of children with negative malaria test results would have enabled comparison of treatment-seeking behaviour among caregivers of children with febrile illnesses irrespective of malaria status and enhanced understanding of general healthcare-seeking behaviour. We have acknowledged this as a limitation in the Discussion section and suggested it as an area for future research. |
| 3. | More than 80% of participants appear to report socially desirable or normatively acceptable characteristics (e.g., monogamy, prompt care-seeking, higher education levels). It would be valuable to discuss whether such a profile is representative of the broader population, especially in rural settings or other regions/ states. Additionally, while the manuscript provides useful insights into family-level dynamics, it lacks key contextual variables such as distance to the health facility and time to receive care upon arrival. | Our study was conducted in a public tertiary hospital located in an urban area, where caregivers are generally more educated and have relatively better access to healthcare than populations in many rural settings. Consequently, the sociodemographic profile of our participants may not fully reflect caregivers in rural communities or other regions of Nigeria, and this has now been acknowledged in the Discussion. Although treatment-seeking behaviour was assessed using a validated interviewer-administered questionnaire, we recognise that socially desirable responses cannot be completely excluded. We have also acknowledged that contextual factors such as distance to the health facility and waiting time before receiving care were not collected and may influence treatment-seeking behaviour. These limitations have been added to the revised manuscript, and we recommend that future studies incorporate health system accessibility variables to provide a more comprehensive understanding of caregivers' treatment-seeking behaviour. |
| 4. | I recommend including the Family APGAR questionnaire and the Perceived Social Support Family Scale (PSS-Fa) in the supplementary materials to enhance readers’ understanding and interpretation of the findings. | The **Family APGAR questionnaire** and the **Perceived Social Support from Family Scale (PSS-Fa)** were included in the questionnaire submitted as supplementary materials. Specifically, both instruments are presented in **Section D of the questionnaire (Supplementary Files S3 and S4).** |
| 5. | Overall, the study is timely and contributes meaningfully to the field. However, the conclusion could be strengthened by providing more explicit policy or programmatic recommendations, particularly regarding interventions to address potential family dysfunction. | We have revised the Conclusion section to include explicit policy and programmatic implications of our findings. Specifically, we now recommend incorporating family functioning assessment into routine paediatric and primary healthcare, strengthening family-centred health education and counselling, and integrating psychosocial support for families with dysfunctional family relationships. These recommendations provide practical strategies for improving caregivers' treatment-seeking behaviour and, ultimately, childhood malaria outcomes. |

**REVIEWER 2**

**REVIEWER’S COMMENTS AND AUTHORS’ CORRECTIONS**

**Thank you so much for your painstaking effort in reviewing our manuscript. Below are the comments to each of the points raised.**

| **S/N** | **REVIEWER’S COMMENTS** | **AUTHORS’ CORRECTION** |
| --- | --- | --- |
| 1. | Has the statistical analysis been performed appropriately and rigorously? Yes, I, however, would recommend more powerful statistical software like R. | We thank you for this valuable suggestion. We agree that R is a powerful and versatile statistical software package. However, the statistical analyses performed in this study were adequately conducted using IBM SPSS Statistics, which is a widely accepted and validated software for epidemiological and clinical research.  We will consider the use of R in future studies. |
| 2. | Is the manuscript presented in an intelligible fashion and written in standard English? Yes, however, it would be advisable if he could operationalize words, e.g., "functional families" and "inappropriate treatment-seeking behavior." | We thank you for this valuable suggestion. We have revised the Methods section by providing clear operational definitions of the key study variables, including **functional family, dysfunctional family, high and low perceived family support**, and **appropriate and inappropriate treatment-seeking behaviour.** These definitions are based on the validated instruments and predefined criteria used in this study, thereby improving the clarity and reproducibility of the findings. |
