# Supplementary material for: Pattern of family dynamics and treatment-seeking behaviour of caregivers of under-five children with uncomplicated malaria in a tertiary hospital in Ilesa, southwestern Nigeria
Source: PLoS One. 2026 Jul 24;21(7):e0354019. doi: 10.1371/journal.pone.0354019 (PMC13399444; doi:10.1371/journal.pone.0354019)
Supplement: Peer review history

# Peer review: Decision Letter 0

**DOI:** 10.1371/journal.pone.0354019.r001

**Authors:** David Diemert

8 Jun 2026

PONE-D-26-12548

Pattern of family dynamics and treatment-seeking behaviour of caregivers of under-five children with uncomplicated malaria in a tertiary hospital in Ilesa, southwestern Nigeria

PLOS One

Dear Dr. Ahmed,

Thank you for submitting your manuscript to PLOS ONE. After careful consideration, we feel that it has merit but does not fully meet PLOS ONE’s publication criteria as it currently stands. Therefore, we invite you to submit a revised version of the manuscript that addresses the points raised during the review process.

Please submit your revised manuscript by Jul 23 2026 11:59PM. If you will need more time than this to complete your revisions, please reply to this message or contact the journal office at plosone@plos.org. When you're ready to submit your revision, log on to https://www.editorialmanager.com/pone/ and select the 'Submissions Needing Revision' folder to locate your manuscript file.

Please include the following items when submitting your revised manuscript:

- A letter that responds to each point raised by the academic editor and reviewer(s). You should upload this letter as a separate file labeled 'Response to Reviewers'.
- A marked-up copy of your manuscript that highlights changes made to the original version. You should upload this as a separate file labeled 'Revised Manuscript with Track Changes'.
- An unmarked version of your revised paper without tracked changes. You should upload this as a separate file labeled 'Manuscript'.

If you would like to make changes to your financial disclosure, please include your updated statement in your cover letter. Guidelines for resubmitting your figure files are available below the reviewer comments at the end of this letter.

If applicable, we recommend that you deposit your laboratory protocols in protocols.io to enhance the reproducibility of your results. Protocols.io assigns your protocol its own identifier (DOI) so that it can be cited independently in the future. For instructions see: https://journals.plos.org/plosone/s/submission-guidelines#loc-laboratory-protocols. Additionally, PLOS ONE offers an option for publishing peer-reviewed Lab Protocol articles, which describe protocols hosted on protocols.io. Read more information on sharing protocols at https://plos.org/protocols?utm_medium=editorial-email&utm_source=authorletters&utm_campaign=protocols.

As the corresponding author, your ORCID iD is verified in the submission system and will appear in the published article. PLOS supports the use of ORCID, and we encourage all coauthors to register for an ORCID iD and use it as well. Please encourage your coauthors to verify their ORCID iD within the submission system before final acceptance, as unverified ORCID iDs will not appear in the published article. *Only*  the individual author can complete the verification step; PLOS staff *cannot*  verify ORCID iDs on behalf of authors.

We look forward to receiving your revised manuscript.

Kind regards,

David J. Diemert, M.D.

Academic Editor

PLOS One

Journal Requirements:

When submitting your revision, we need you to address these additional requirements.

1. Please ensure that your manuscript meets PLOS ONE's style requirements, including those for file naming. The PLOS ONE style templates can be found at

https://journals.plos.org/plosone/s/file?id=wjVg/PLOSOne_formatting_sample_main_body.pdf and https://journals.plos.org/plosone/s/file?id=ba62/PLOSOne_formatting_sample_title_authors_affiliations.pdf

2. Your ethics statement should only appear in the Methods section of your manuscript. If your ethics statement is written in any section besides the Methods, please delete it from any other section.

3. Please amend either the title on the online submission form (via Edit Submission) or the title in the manuscript so that they are identical.

4. Please update your submission to use the PLOS LaTeX template. The template and more information on our requirements for LaTeX submissions can be found at http://journals.plos.org/plosone/s/latex.

5. We note that Figure S2 in your submission contain copyrighted images. All PLOS content is published under the Creative Commons Attribution License (CC BY 4.0), which means that the manuscript, images, and Supporting Information files will be freely available online, and any third party is permitted to access, download, copy, distribute, and use these materials in any way, even commercially, with proper attribution. For more information, see our copyright guidelines: http://journals.plos.org/plosone/s/licenses-and-copyright.

We require you to either (1) present written permission from the copyright holder to publish these figures specifically under the CC BY 4.0 license, or (2) remove the figures from your submission:

a. You may seek permission from the original copyright holder of Figure S2 to publish the content specifically under the CC BY 4.0 license.

We recommend that you contact the original copyright holder with the Content Permission Form (http://journals.plos.org/plosone/s/file?id=7c09/content-permission-form.pdf) and the following text:

“I request permission for the open-access journal PLOS ONE to publish XXX under the Creative Commons Attribution License (CCAL) CC BY 4.0 (http://creativecommons.org/licenses/by/4.0/). Please be aware that this license allows unrestricted use and distribution, even commercially, by third parties. Please reply and provide explicit written permission to publish XXX under a CC BY license and complete the attached form.”

Please upload the completed Content Permission Form or other proof of granted permissions as an "Other" file with your submission.

In the figure caption of the copyrighted figure, please include the following text: “Reprinted from [ref] under a CC BY license, with permission from [name of publisher], original copyright [original copyright year].”

b. If you are unable to obtain permission from the original copyright holder to publish these figures under the CC BY 4.0 license or if the copyright holder’s requirements are incompatible with the CC BY 4.0 license, please either i) remove the figure or ii) supply a replacement figure that complies with the CC BY 4.0 license. Please check copyright information on all replacement figures and update the figure caption with source information. If applicable, please specify in the figure caption text when a figure is similar but not identical to the original image and is therefore for illustrative purposes only.

6. We notice that your supplementary [figures] are included in the manuscript file. Please remove them and upload them with the file type 'Supporting Information'. Please ensure that each Supporting Information file has a legend listed in the manuscript after the references list.

7. Please include captions for your Supporting Information files at the end of your manuscript, and update any in-text citations to match accordingly. Please see our Supporting Information guidelines for more information: http://journals.plos.org/plosone/s/supporting-information.

8. We are unable to open your Supporting Information file [File Name]. Please kindly revise as necessary and re-upload.

9. Thank you for providing your underlying data as Supporting Information.

We note that the data set contains text or data that is not in English. Please note that PLOS is an English-language publisher, so we require data sets to be provided in English as well. Please upload an English-language version of your data set.

This will also allow us to determine if your data follows PLOS standards per our Data Availability policy here: https://journals.plos.org/plosone/s/data-availability

10. If the reviewer comments include a recommendation to cite specific previously published works, please review and evaluate these publications to determine whether they are relevant and should be cited. There is no requirement to cite these works unless the editor has indicated otherwise.

11. Please review your reference list to ensure that it is complete and correct. If you have cited papers that have been retracted, please include the rationale for doing so in the manuscript text, or remove these references and replace them with relevant current references. Any changes to the reference list should be mentioned in the rebuttal letter that accompanies your revised manuscript. If you need to cite a retracted article, indicate the article’s retracted status in the References list and also include a citation and full reference for the retraction notice.

[Note: HTML markup is below. Please do not edit.]

Reviewers' comments:

Reviewer's Responses to Questions

**Comments to the Author**

1. Is the manuscript technically sound, and do the data support the conclusions?

The manuscript must describe a technically sound piece of scientific research with data that supports the conclusions. Experiments must have been conducted rigorously, with appropriate controls, replication, and sample sizes. The conclusions must be drawn appropriately based on the data presented.

Reviewer #1: Yes

Reviewer #2: Yes

**********

2. Has the statistical analysis been performed appropriately and rigorously?

Reviewer #1: Yes

Reviewer #2: Yes

**********

3. Have the authors made all data underlying the findings in their manuscript fully available?

The PLOS Data policy requires authors to make all data underlying the findings described in their manuscript fully available without restriction, with rare exception (please refer to the Data Availability Statement in the manuscript PDF file). The data should be provided as part of the manuscript or its supporting information, or deposited to a public repository. For example, in addition to summary statistics, the data points behind means, medians and variance measures should be available. If there are restrictions on publicly sharing data—e.g. participant privacy or use of data from a third party—those must be specified.

Reviewer #1: Yes

Reviewer #2: Yes

**********

4. Is the manuscript presented in an intelligible fashion and written in standard English?

PLOS ONE does not copyedit accepted manuscripts, so the language in submitted articles must be clear, correct, and unambiguous. Any typographical or grammatical errors should be corrected at revision, so please note any specific errors here.

Reviewer #1: Yes

Reviewer #2: Yes

**********

5. Review Comments to the Author

Please use the space provided to explain your answers to the questions above. You may also include additional comments for the author, including concerns about dual publication, research ethics, or publication ethics. (Please upload your review as an attachment if it exceeds 20,000 characters)

Reviewer #1: The manuscript is well written, employs an appropriate methodological approach, and addresses an important yet often overlooked aspect of health-seeking behavior. I have only a few minor comments for the authors to consider:

1. The study setting is not sufficiently described. It would be helpful to clarify whether the health facility is located in a rural or urban area, is the area high endemic or low, and whether it is publicly or privately owned. Also how malaria treatment service is provided-either freely accessible or with some consultation fee etc...(also patient trajectory in the event a referal is needed..if such health system info is available).

2. The study population includes only participants with a positive malaria test. The authors may wish to discuss how the findings might differ if individuals with negative test results were also included (e.g., as a test-negative comparison group), particularly in relation to general health-seeking behavior.

3. More than 80% of participants appear to report socially desirable or normatively acceptable characteristics (e.g., monogamy, prompt care-seeking, higher education levels). It would be valuable to discuss whether such a profile is representative of the broader population, especially in rural settings or other regions/ states. Additionally, while the manuscript provides useful insights into family-level dynamics, it lacks key contextual variables such as distance to the health facility and time to receive care upon arrival.

4. I recommend including the Family APGAR questionnaire and the Perceived Social Support Family Scale (PSS-Fa) in the supplementary materials to enhance readers’ understanding and interpretation of the findings.

Overall, the study is timely and contributes meaningfully to the field. However, the conclusion could be strengthened by providing more explicit policy or programmatic recommendations, particularly regarding interventions to address potential family dysfunction.

Reviewer #2: 1. Is the manuscript technically sound, and do the data support the conclusions?

Yes, the sample size was well calculated, and the author even added about 10% for attrition and gave the paper statistical power.

2. Has the statistical analysis been performed appropriately and rigorously?

Yes, I, however, would recommend more powerful statistical software like R.

3. Is the manuscript presented in an intelligible fashion and written in standard English?

Yes, however, it would be advisable if he could operationalize words, e.g., "functional families" and "inappropriate treatment-seeking behavior."

**********

6. PLOS authors have the option to publish the peer review history of their article (what does this mean?). If published, this will include your full peer review and any attached files.

If you choose “no”, your identity will remain anonymous but your review may still be made public.

**Do you want your identity to be public for this peer review?**  For information about this choice, including consent withdrawal, please see our Privacy Policy.

Reviewer #1: **Yes:**  Geofrey Makenga

Reviewer #2: **Yes:**  Brian Seda

**********

[NOTE: If reviewer comments were submitted as an attachment file, they will be attached to this email and accessible via the submission site. Please log into your account, locate the manuscript record, and check for the action link "View Attachments". If this link does not appear, there are no attachment files.]

To ensure your figures meet our technical requirements, please review our figure guidelines: https://journals.plos.org/plosone/s/figures

You may also use PLOS’s free figure tool, NAAS, to help you prepare publication quality figures: https://journals.plos.org/plosone/s/figures#loc-tools-for-figure-preparation.

NAAS will assess whether your figures meet our technical requirements by comparing each figure against our figure specifications.

# Author comment: Author response to Decision Letter 1

**DOI:** 10.1371/journal.pone.0354019.r002

1 Jul 2026

REVIEWER’S COMMENTS AND AUTHORS’ CORRECTIONS

Thank you so much for your painstaking effort in reviewing our manuscript. Below are the comments to each of the points raised.

SECTION REVIEWER’S COMMENTS AUTHORS’ CORRECTION

1. The study setting is not sufficiently described. It would be helpful to clarify whether the health facility is located in a rural or urban area, is the area high endemic or low, and whether it is publicly or privately owned.

Also how malaria treatment service is provided-either freely accessible or with some consultation fee etc...(also patient trajectory in the event a referal is needed..if such health system info is available).

Thank you for this valuable suggestion. We have expanded the description of the study setting in the Methods section to include the location of the hospital (urban), malaria endemicity of the study area (stable high transmission), ownership of the hospital (public tertiary hospital under Obafemi Awolowo University Teaching Hospitals Complex), the financing of malaria services (fee-for-service with health insurance coverage for some patients), and the referral pathway for children requiring higher levels of care. These additions provide better context for readers regarding the healthcare system within which the study was conducted.

2. The study population includes only participants with a positive malaria test. The authors may wish to discuss how the findings might differ if individuals with negative test results were also included (e.g., as a test-negative comparison group), particularly in relation to general health-seeking behavior.

The objective of our study was to investigate the relationship between family dynamics and treatment-seeking behaviour among caregivers of under-five children with laboratory-confirmed uncomplicated malaria. Consequently, only children with parasitologically confirmed malaria were included to ensure a homogeneous study population and to minimise outcome misclassification. We agree that inclusion of children with negative malaria test results would have enabled comparison of treatment-seeking behaviour among caregivers of children with febrile illnesses irrespective of malaria status and enhanced understanding of general healthcare-seeking behaviour. We have acknowledged this as a limitation in the Discussion section and suggested it as an area for future research.

3. More than 80% of participants appear to report socially desirable or normatively acceptable characteristics (e.g., monogamy, prompt care-seeking, higher education levels). It would be valuable to discuss whether such a profile is representative of the broader population, especially in rural settings or other regions/ states. Additionally, while the manuscript provides useful insights into family-level dynamics, it lacks key contextual variables such as distance to the health facility and time to receive care upon arrival. Our study was conducted in a public tertiary hospital located in an urban area, where caregivers are generally more educated and have relatively better access to healthcare than populations in many rural settings. Consequently, the sociodemographic profile of our participants may not fully reflect caregivers in rural communities or other regions of Nigeria, and this has now been acknowledged in the Discussion. Although treatment-seeking behaviour was assessed using a validated interviewer-administered questionnaire, we recognise that socially desirable responses cannot be completely excluded. We have also acknowledged that contextual factors such as distance to the health facility and waiting time before receiving care were not collected and may influence treatment-seeking behaviour. These limitations have been added to the revised manuscript, and we recommend that future studies incorporate health system accessibility variables to provide a more comprehensive understanding of caregivers' treatment-seeking behaviour.

4. I recommend including the Family APGAR questionnaire and the Perceived Social Support Family Scale (PSS-Fa) in the supplementary materials to enhance readers’ understanding and interpretation of the findings. The Family APGAR questionnaire and the Perceived Social Support from Family Scale (PSS-Fa) were included in the questionnaire submitted as supplementary materials. Specifically, both instruments are presented in Section D of the questionnaire (Supplementary Files S3 and S4).

5. Overall, the study is timely and contributes meaningfully to the field. However, the conclusion could be strengthened by providing more explicit policy or programmatic recommendations, particularly regarding interventions to address potential family dysfunction. We have revised the Conclusion section to include explicit policy and programmatic implications of our findings. Specifically, we now recommend incorporating family functioning assessment into routine paediatric and primary healthcare, strengthening family-centred health education and counselling, and integrating psychosocial support for families with dysfunctional family relationships. These recommendations provide practical strategies for improving caregivers' treatment-seeking behaviour and, ultimately, childhood malaria outcomes.

Attachment Submitted filename: Response to Reviewers.docx

# Editor report: Decision Letter 1

**DOI:** 10.1371/journal.pone.0354019.r003

**Authors:** David Diemert

2 Jul 2026

Pattern of family dynamics and treatment-seeking behaviour of caregivers of under-five children with uncomplicated malaria in a tertiary hospital in Ilesa, southwestern Nigeria

PONE-D-26-12548R1

Dear Dr. Ahmed,

We’re pleased to inform you that your manuscript has been judged scientifically suitable for publication and will be formally accepted for publication once it meets all outstanding technical requirements.

Within one week, you’ll receive an e-mail detailing the required amendments. When these have been addressed, you’ll receive a formal acceptance letter and your manuscript will be scheduled for publication.

An invoice will be generated when your article is formally accepted. Please note, if your institution has a publishing partnership with PLOS and your article meets the relevant criteria, all or part of your publication costs will be covered. Please make sure your user information is up-to-date by logging into Editorial Manager at Editorial Manager® and clicking the ‘Update My Information' link at the top of the page. For questions related to billing, please contact billing support.

If your institution or institutions have a press office, please notify them about your upcoming paper to help maximize its impact. If they’ll be preparing press materials, please inform our press team as soon as possible -- no later than 48 hours after receiving the formal acceptance. Your manuscript will remain under strict press embargo until 2 pm Eastern Time on the date of publication. For more information, please contact onepress@plos.org.

Kind regards,

David J. Diemert, M.D.

Academic Editor

PLOS One

Additional Editor Comments (optional):

Reviewers' comments:

# Editor report: Acceptance letter

**DOI:** 10.1371/journal.pone.0354019.r004

**Authors:** David Diemert

PONE-D-26-12548R1

PLOS One

Dear Dr. Ahmed,

I'm pleased to inform you that your manuscript has been deemed suitable for publication in PLOS One. Congratulations! Your manuscript is now being handed over to our production team.

At this stage, our production department will prepare your paper for publication. This includes ensuring the following:

* All references, tables, and figures are properly cited

* All relevant supporting information is included in the manuscript submission,

* There are no issues that prevent the paper from being properly typeset

You will receive further instructions from the production team, including instructions on how to review your proof when it is ready. Please keep in mind that we are working through a large volume of accepted articles, so please give us a few days to review your paper and let you know the next and final steps.

Lastly, if your institution or institutions have a press office, please let them know about your upcoming paper now to help maximize its impact. If they'll be preparing press materials, please inform our press team within the next 48 hours. Your manuscript will remain under strict press embargo until 2 pm Eastern Time on the date of publication. For more information, please contact onepress@plos.org.

You will receive an invoice from PLOS for your publication fee after your manuscript has reached the completed accept phase. If you receive an email requesting payment before acceptance or for any other service, this may be a phishing scheme. Learn how to identify phishing emails and protect your accounts at https://explore.plos.org/phishing.

If we can help with anything else, please email us at customercare@plos.org.

Thank you for submitting your work to PLOS ONE and supporting open access.

Kind regards,

PLOS ONE Editorial Office Staff

on behalf of

Dr. David J. Diemert

Academic Editor

PLOS One
